# From Deutsche Zeitschrift to International journal of legal medicine—100 years of legal medicine through the lens of journal articles, Part 4: International journal of legal medicine from 1990 to 2022

**DOI:** 10.1007/s00414-023-03107-w

**Published:** 2023-10-16

**Authors:** Tony Fracasso, Ingo Wirth, Heidi Pfeiffer, Andreas Schmeling

**Affiliations:** 1 University Center of Legal Medicine, Geneva University Hospitals, Geneva, Switzerland; 2 Berlin, Germany; 3 Institute of Legal Medicine, University Hospital Münster, Münster, Germany

**Keywords:** History of medicine, Legal medicine, Official publications, Academic articles

## Abstract

This is the fourth and final paper in a series related to the analysis of articles published in this journal during its first 100 years of activity. This article covers the time span from 1990 to 2022. It is important to note that, given the period covered by this analysis, it does not aim to provide a historical overview but rather an examination of the most recent trends in our discipline compared to the past. Between 1990 (Volume 104) and 2022 (Volume 136), 4004 articles were published in the International Journal of Legal Medicine (IJLM) across 33 volumes. This corresponds to 53% of all the articles published since the launch of the journal. When compared to the period from 1970 to 1990, some categories no longer appear to be as relevant (e.g., sexual medicine, 1 article; social medicine, 0 articles; biography, 3 articles; history, 4 articles). Conversely, the most recent period has shown an increasing importance in forensic genetics (1388 articles) and the emergence of new significant topics that merit their own classification, such as age estimation (286 articles), forensic anthropology (189 articles), forensic imaging (150 articles), and forensic entomology (90 articles).

**Supplementary Information:**

The online version contains supplementary material available at 10.1007/s00414-023-03107-w.

## Introduction

This article delves into the publications of the International Journal of Legal Medicine (IJLM) from 1990 to 2022, encompassing volumes 104 to 136. Volume 104 commenced in December 1990, leading to an overlap with the previously analyzed period (1970–1990), as 1990 was the last year in which the Deutsche Zeitschrift was published. Over these thirty-three years, the journal underwent a significant transformation, evolving from a prestigious regional German-speaking reference to a globally renowned international publication in the field of Legal Medicine and related sciences. The pivotal decision by the Editorial board, led by Bernd Brinkmann, to convert the journal into an exclusively English-speaking publication [1], marked the inception of this transformation. While historical perspectives could be drawn for the earlier parts of this series covering the time periods 1922–1944, 1948–1969, and 1970–1990 [2–4], the present part focusing on the last three decades can primarily offer an analysis of the most recent trends in our discipline when compared to the past.

To conduct this analysis, SpringerNature's Editorial team provided invaluable information on the journal's development in terms of metrics. Table 1 presents the top ten most cited articles during this analysis period (the list of the one hundred most cited papers can be found in Table 1 ESM). It is noteworthy that among the top ten, five articles relate to forensic genetics, three to age estimation, one to entomology, and only one can be classified as classical forensic pathology (related to COVID-19): a revolution has taken place.

**Table 1:** The ten most cited articles published in the International Journal of Legal Medicine in the period 1990—2022. Source Clarivate™

1) Kayser, M; Caglia, A; Corach, D; Fretwell, N; Gehrig, C; Graziosi, G; Heidorn, F; Herrmann, S; Herzog, B; Hidding, M; Honda, K; Jobling, M; Krawczak, M; Leim, K; Meuser, S; Meyer, E; Oesterreich, W; Pandya, A; Parson, W; Penacino, G; Perez Lezaun, A; Piccinini, A; Prinz, M; Schmitt, C; Schneider, PM; Szibor, R; Teifel Greding, J; Weichold, G; deKnijff, P; Roewer, L (1997) Evaluation of Y-chromosomal STRs: A multicenter study. DOI 10.1007/s004140050051. **Cited 579 times**
2) Amendt, J; Campobasso, CP; Gaudry, E; Reiter, C; LeBlanc, HN; Hall, MJR (2007) Best practice in forensic entomology—standards and guidelines. DOI 10.1007/s00414-006-0086-x. **Cited 460 times**
3) Schmeling, A; Grundmann, C; Fuhrmann, A; Kaatsch, HJ; Knell, B; Ramsthaler, F; Reisinger, W; Riepert, T; Ritz-Timme, S; Roesing, FW; Roetzscher, K; Geserick, G (2008) Criteria for age estimation in living individuals. DOI 10.1007/s00414-008-0254-2. **Cited 391 times**
4) Ritz-Timme, S; Cattaneo, C; Collins, MJ; Waite, ER; Schutz, HW; Kaatsch, HJ; Borrman, HIM (2000) Age estimation: The state of the art in relation to the specific demands of forensic practise. DOI 10.1007/s004140050283. **Cited 325 times**
5) Bar, W; Brinkmann, B; Budowle, B; Carracedo, A; Gill, P; Lincoln, P; Mayr, W; Olaisen, B (1997) DNA recommendations—Further report of the DNA Commission of the ISFH regarding the use of short tandem repeat systems. DOI 10.1007/s004140050061. **Cited 311 times**
6) Jobling, MA; Pandya, A; TylerSmith, C (1997) The Y chromosome in forensic analysis and paternity testing. DO 10.1007/s004140050050. **Cited 291 times**
7) Edler, C; Schroeder, A S; Aepfelbacher, M; Fitzek, A; Heinemann, A; Heinrich, F; Klein, A; Langenwalder, F; Luetgehetmann, M; Meissner, K; Pueschel, K; Schaedler, J; Steurer, S; Mushumba, H; Sperhake, JP (2020) Dying with SARS-CoV-2 infection-an autopsy study of the first consecutive 80 cases in Hamburg, Germany. DOI 10.1007/s00414-020-02317-w. **Cited 279 times**
8) Wislon, MR; Dizinno, JA; Polanskey, D; Replogle, J; Budowle, B (1995) Validation of mitochondrial-DNA sequencing for forensic casework analysis. DOI 10.1007/BF01369907. **Cited 275 times**
9) deKnijff, P; Kayser, M; Caglia, A; Corach, D; Fretwell, N; Gehrig, C; Graziosi, G; Heidorn, F; Herrmann, S; Herzog, B; Hidding, M; Honda, K; Jobling, M; Krawczak, M; Leim, K; Meuser, S; Meyer, E; Oesterreich, W; Pandya, A; Parson, W; Penacino, G; PerezLezaun, A; Piccinini, A; Prinz, M; Schmitt, C; Schneider, PM; Szibor, R; Teifel Greding, J; Weichhold, G; Roewer, L (1997) Chromosome Y microsatellites: Population genetic and evolutionary aspects. DOI 10.1007/s004140050052. **Cited 249 times**
10) Schmeling, A; Schulz, R; Reisinger, W; Muhler, M; Wernecke, KD; Geserick, G (2004) Studies on the time frame for ossification of the medial clavicular epiphyseal cartilage in conventional radiography. DOI 10.1007/s00414-003-0404-5. **Cited 243 times**

## Analysis of academic articles

From 1990 to 2022, the IJLM published four thousand and four articles across thirty-three volumes. In the last thzirty-three years of its centenary existence, the journal published 53% of all articles. The distribution of categories/topics is outlined in Table 2. In comparison to the period from 1970 to 1990, certain categories no longer maintain their relevance (e.g., sexual medicine, one article; social medicine, no articles; biography, two articles; history, four articles). Conversely, the recent era witnessed a growing importance of forensic genetics (one thousand three hundred eighty-eight articles) as well as the emergence of new pertinent topics that warrant their own classification, such as age estimation (two hundred eighty six articles), forensic anthropology (one hundred eighty-nine articles), forensic imaging (one hundred fifty articles), and forensic entomology (ninety articles). Table 3 illustrates the comparison with the previously investigated periods.

**Table 2:** Distribution of the papers published between 1990 and 2022 into the different categories

Legal issues, expert witnesses, insurance medicine	12
General legal medicine	250
Forensic traumatology and pathology	778
Forensic toxicology	348
Identification of unknown decedents	54
Forensic genetics	1388
Scientific technical criminalistics	132
Clinical legal medicine	49
Forensic psychiatry and psychology	3
Sexual medicine	1
Traffic medicine	20
Social medicine	0
Criminology	6
Age estimation	286
Forensic Anthropology	189
Forensic Imaging	150
Forensic Entomology	90
Editorials and comments	41
Letters to the editor	157
Book reviews	43
Total	4004

**Table 3:** Comparison of the distribution of the articles in the different sub-discipline in the four periods (%)

Sub-discipline	1922–1944	1948–1969	1970–1990	1991–2022
History and evolution of the discipline	1,6	0,5	0,6	0,1
Personalia	1,9	2,7	0,7	0,1
Legal issues, expert witnesses, insurance medicine	7,9	3,9	3,7	0,3
General legal medicine	10,3	9,6	14,4	6,6
Forensic traumatology and pathology	32,2	28,2	27,8	20,7
Forensic toxicology	16,6	25,3	19,6	9,2
Identification of unknown bodies	1,2	2,1	2,7	1,4
Forensic genetics	8,5	15,9	23,5	36,9
Scientific technical criminalistics	6,4	1,2	1,7	3,5
Clinical legal medicine	0,4	0,6	0,1	1,2
Forensic psychiatry and psychology	4,3	2,6	0,6	0,1
Sexual medicine	3,2	1,4	0,1	0
Traffic medicine	1,1	4,2	3,1	0,6
Social medicine	0,6	0,6	0,1	0
Criminology	3,8	1,2	1,3	0,2
Age estimation	nc	nc	nc	7,6
Forensic Anthropology	nc	nc	nc	5,1
Forensic Imaging	nc	nc	nc	4
Forensic Entomology	nc	nc	nc	2,4
Total	100	100	100	100

## History and evolution of the discipline

This category was notably underrepresented during this period, with only four publications. One article (125/393)^*^ serves as an overview of the bibliometric evolution of biomedico-legal research in Europe between 2005 and 2010. Two articles were published in the same volume as a short series discussing the evolution of autopsy and histology techniques (131/1069) as well as the development of other "ancillary" investigations that gradually evolved into "autonomous" sciences, such as forensic genetics, omics, and imaging (131/1085). The last article attributable to this category is the first part of the present series (136/1897) [2].

## Personalia

The observed decreasing trend in the publication of personalia between 1970 and 1990 was reaffirmed between 1990 and 2022. During this later period, the journal published only three articles attributed to this category, which comprised obituaries for Karl Sellier (110/239), Bruno Maria Altamura (117/N1), and Otto Prokop (123/449).

## Legal issues, expert activity, insurance medicine

This category encompasses twelve articles. Among them, six publications focused on insurance medicine and damage assessment within a civil context. Specifically, four articles addressed damage assessment after whiplash injury (118/235, 121/337, 130/13, 135/1637), while two manuscripts proposed guidelines and evaluation criteria for personal injury and damage (130/1) and for psychic and existential damage (130/1387). A group from Frankfurt explored the influence of religions on attitudes towards brain death, organ transplantation, and autopsy (134/1203). A Swiss group discussed the ethical and legal aspects of creating a body farm (134/1875) and a forensic pathology biobank in Switzerland (136/919). Another publication from Spain described the judicial consequences of completing the medical death certificate (136/365). One particularly original publication (136/963) proposed the use of model figurines during expert opinion. Finally, publication 136/1027 discussed the possibility of adopting the Bayesian approach for forensic pathology opinions.

## General legal medicine

This category comprised two hundred fifty articles. Among them, sixty-nine articles focused on the estimation of the time since death. Besides investigations into temperature-based approaches (113/320, 113/303, 119/185, 121/82, 121/267, 125/437, 125/503, 126/79, 130/1243, 132/781, 133/491, 134/591, 135/1669, 135/2479, 136/1341), articles proposed alternative methods on protein degradation (123/305, 130/421, 130/433, 130/447, 130/1547, 131/479, 131/1615, 132/1349, 133/899, 134/1775, 135/837, 135/837, 135/1627, 135/2081), imaging (130/1003, 131/699, 132/263, 132/269, 135/2615), biochemistry (135/269, 135/845), RNA (127/573, 130/615, 133/1629, 134/149), microbiology (129/623, 135/223), apoptosis (135/539). Due to the development of forensic anthropology into a specific sub-discipline over the last three decades, articles concerning the estimation of the time since death in bone remains were included in this category.

Livor mortis was the topic of six articles (106/91, 106/209, 113/81, 119/355, 122/91, 130/1599), rigor mortis was addressed in seven articles (112/167, 113/240, 127/127, 127/971, 131/1039, 133/1133, 134/1939), and twenty-four articles discussed late signs of death (106/225, 111/35, 112/2, 117/102, 118/206, 125/643,126/3, 127/437, 127/975, 128/379, 128/725, 128/719, 128/795, 128/1039, 129/641, 129/661, 130/281, 130/253, 131/1141, 132/301, 132/649, 134/793, 136/887, 136/1201).

Three articles presented different approaches to educating medical students and physicians on external examination (125/857, 131/1701, 132/311). Autopsy techniques, including special sampling techniques, were reported in eight articles (111/157, 111/208, 125/587, 129/307, 130/1033, 133/1477, 133/1809, 132/741). Article 128/1031 was related to the education of medical students on autopsy. Bernd Brinkmann’s editorial on the harmonization of medicolegal autopsy rules (113/1) was also listed in this category.

Thirty-one articles focused on the topic of signs of vitality, with twenty addressing local vitality (105/203, 105/223, 106/145, 106/312, 107/257, 109/130, 111/251, 117/14, 117/356, 118/282, 125/836, 126/957, 127/957, 128/319, 128/321, 129/537, 132/1375, 135/1837, 135/1843, 135/2537), and eleven on general vitality (112/101, 112/195, 124/583, 125/403, 128/967, 128/317, 129/313, 130/1281, 131/1707, 134/1159, 135/903).

Sixty-one articles dealt with wound age estimation, with twenty-one articles presenting results from animal experiments on mice or rats (108/231, 111/6, 111/10, 111/251, 113/244, 113/1623, 116/267, 117/46, 119/16, 124/27, 124/397, 126/113, 125/549, 126/807, 128/779, 130/163, 131/691, 134/273, 134/2177, 136/149, 136/1781), and thirty-four on human samples (105/21, 105/93, 105/99, 105/169, 105/325, 105/329, 106/31, 106/35, 107/29, 109/29, 113/70, 113/288, 115/61, 118/32, 107/60, 107/197, 108/262, 110/240, 110/299, 111/169, 112/159, 112/249, 114/338, 116/87, 118/320, 121/175, 122/143, 122/409, 123/299, 128/403, 129/1049, 129/1043, 132/531, 134/597). The investigations explored various methods, including special histology staining (i.e., 107/60, 108/262), immunohistochemical investigations, and more recently RNA-based approaches (i.e., 134/2177).

## Forensic traumatology and pathology

A total of seven hundred seventy-eight articles fall under this category. One hundred thirteen of these articles focused on blunt force injuries, while fifty-two were related to traumatic brain injury (104/49, 104/209, 105/243, 106/152, 107/69, 107/99, 107/326, 110/305, 112/227, 112/261, 113/70, 113/221, 114/338, 115/121, 116/92, 117/153, 118/32, 119/177, 120/380, 121/223, 121/365, 122/337, 122/359, 123/189, 125/587, 126/467, 126/835, 127/103, 127/159, 129/105, 129/701, 129/1085, 130/771, 132/1719, 133/539, 133/871, 133/1107, 133/1603, 134/295, 134/2167, 134/2187, 135/235, 135/1525, 135/1481, 135/1525, 135/2323, 136/591, 136/871, 136/1009, 136/1321, 136/1621, 136/1841). Road traffic accidents accounted for twenty articles (104/355, 105/11, 105/121, 106/41, 111/85, 113/84, 114/316, 115/165, 117/226, 120/246, 122/511, 130/463, 130/1593, 131/1023, 132/1729, 133/547, 134/1403, 134/1431, 135/565, 135/893), while nine were related to fall-related traumas (120/212, 125/1, 127/11, 132/205, 132/771, 132/1699, 133/847, 135/245, 135/527), and seven to CPR-related lesions (106/215, 109/84, 111/93, 129/1035, 130/1581, 131/1655, 132/1733).

Sharp force injuries were the primary focus in twenty-one articles (109/01, 110/267, 112/313, 113/107, 113/259, 114/346, 115/167, 118/188, 118/348, 119/47, 119/226, 120/369, 122/179, 122/281, 131/1313, 133/1429, 134/1791, 135/301, 135/313, 135/555, 135/2683), a consistent part of which were case reports. Among these articles, those by Karger et al. concerning self-infliction, diagnostic criteria for suicide versus homicide, and the potential for physical activity after fatal injury deserve mention (110/267, 112/188, 113/259). Papers describing experimental models in the fields of sharp forces have been counted in the section Scientific and Technical Criminalistics.

Asphyxia was the subject of forty-three articles. The pathophysiology of asphyxia was the main topic in thirteen articles (104/313, 106/258, 106/156, 106/281, 109/163, 112/351, 113/268, 124/559, 128/117, 130/153, 132/655, 136/133, 136/1091). Case reports or case series were reported in twenty papers (104/47, 108/140, 110/164, 112/55, 118/106, 119/98, 120/110, 122/499, 123/517, 125/289, 125/459, 126/765, 129/1103, 129/1109, 133/177, 134/1073, 134/1465, 135/347, 136/1359, 136/1773).

Drowning (including diatom tests) was discussed in thirty-five papers, twenty-one of which describe diagnostic methods involving the detection of diatoms or aquatic bacteria (107/37, 108/39, 108/323, 111/229, 112/163, 114/6, 127/459, 130/1303, 131/1573, 132/487, 132/1611, 133/1351, 133/1819, 134/1037, 134/1375, 134/2149, 135/497, 135/817, 135/2519, 136/911, 136/1745).

Gunshot wounds were reported and discussed in ninety papers. Characteristics of entrance and/or exit wounds were reported in nine articles (116/262, 117/19, 119/217, 120/257, 121/105, 127/931, 131/441, 136/629, 136/1597). Gunshot residue was discussed in nine papers (116/1, 118/343, 121/287, 126/525, 129/819, 130/1045, 133/169, 134/2195). Multiple gunshot suicides were presented in three papers (133/1469, 110/188, 136/179). Karger et al. proposed criteria for self-infliction (110/33, 116/273) and discussed the topic of incapacitation (108/53, 108/117). A crossbow was the weapon used in four reports (111/88, 112/58, 118/332, 134/283). Seven articles were related to explosive devices (105/35, 112/372, 114/103, 125/473, 127/225, 131/1581, 133/565). Papers describing experimental models in ballistics have been counted in the section Scientific and Technical Criminalistics.

Three case reports (111/331, 120/36, 134/1785) and two case series (134/1353, 131/677) presented cases of electrocution. Fineschi et al. described morphology changes of the myocardium in twenty-one cases of death by electrocution (120/79). Guangtao et al. investigated the hand-to-foot circuit pathway in a rat model (131/433). Xin et al. described the state of the art in the forensic diagnosis of electrocution, without typical electric marks (135/2469).

Twenty-three papers could be ascribed to the topic of heat exposure. Four studies investigated the immunohistochemical expression of heat shock proteins, Tau protein, and ubiquitin in various organs and tissues (120/355, 128/ 967). Two studies explored the biochemistry of heat exposure (125/11, 127/93). Recently, an article by Bernitz et al. on tongue protrusion as an indicator of vital burning (128/309) was the starting point of a debate about the significance of this common finding (128/317, 128/319, 128/321, 129/313, 129/315, 130/1253, 133/1279).

Cold exposure and hypothermia were the main topics in fifteen articles. Classical signs and typical behaviors were frequently discussed, including muscle hemorrhages (131/1423), the inner knee sign (126/415), Wischnewsky spots (131/1639), the hide and die syndrome (107/250, 108/116), and paradoxical undressing (131/1341, 132/1111). Biochemistry was the investigative method discussed in three papers (125/11, 127/267, 129/2899), while more recently, new methods have been explored in this context, such as mRNA expression (133/335) or metabolomics (133/889).

Among the articles related to sudden death from natural disease in adults, papers on sudden death from cardiovascular origin predominated. Numerous groups tried to develop investigation strategies to detect cardiac ischemia as early as possible (e.g., 106/135, 113/215, 115/142, 130/915, 130/1265, 132/197, 132/425, 132/1339, 133/529, 136/159). Other authors have proposed diagnostic approaches (e.g., 131/393, 135/483, 135/1555).

Immunohistochemistry (114/291, 133/338) and biochemistry (119/80, 127/799, 130/1035, 132/1685) have been proposed as investigative methods for the diagnosis of sepsis, with procalcitonin emerging as a particularly valuable marker (114/237, 124/427, 126/505, 126/567, 129/117).

Among the infections that have been reported as potential causes of natural death, Covid-19 disease has a peculiar role with fourteen papers that can be ascribed to this section. Edler et al. (134/1275) reported on a series of eighty autopsies performed in Hamburg in the very early phase of the pandemic. Four case reports described the histopathology of the disease in various organs (134/1271, 134/1285, 134/2205, 135/577). The question of the infectivity of dead bodies and risk exposure for personnel handling infected bodies was investigated in three papers (135/2055, 135/2531, 136/935). Schneider et al. reported on a series of fatalities after Covid-19 vaccination (135/2335).

Sudden Infant Death Syndrome (SIDS) was the main topic in fifty-eight articles. Diagnostic criteria and classification were described in four articles (118/163, 120/331, 126/271, 134/1015). Among the risk factors that have been investigated, the potential role of organ anomalies was described in ten papers (106/244, 106/249, 107/187, 110/63, 110/199, 110/316, 112/31, 113/332, 118/221, 134/2143). The sleeping position was discussed in four papers (112/22, 123/41, 132/181, 132/1389). Infections were investigated in seven papers (104/3, 105/333, 109/219, 107/291, 108/85, 117/237, 119/2029. Genetic variants were reported in seventeen articles (124/301, 127/1087, 128/43, 128/621, 129/977, 129/985, 130/415, 130/1025, 130/1069, 133/863, 134/1639, 135/207, 135/719, 135/1179, 135/1375, 135/1499, 136/1113).

## Forensic toxicology

A total of three hundred forty-eight articles could be attributed to this category. Among them, fifty-four were case reports where the primary focus was on intoxication (104/239, 106/271, 108/268, 110/220, 111/91, 111/205, 111/265, 111/305, 112/62, 112/268, 113/164, 113/171, 114/248, 114/352, 116/54, 116/238, 116/357, 118/310, 119/236, 120/168, 120/241, 121/48, 121/214, 122/503, 122/507, 123/327, 123/387, 126/447, 126/953, 127/85, 128/65, 128/483, 129/487, 129/1247, 130/723, 130/981, 130/1223, 130/1231, 130/1291, 130/1535, 130/1541, 131/1009, 133/133, 134/251, 134/703, 134/1003, 134/2133, 135/175, 135/1455, 136/695, 136/1291, 136/1297, 136/1309, 136/1585). Additionally, twenty articles presented data from large case series (106/5, 112/198, 119/344, 121/409, 122/395, 123/109, 123/363, 124/1, 124/381, 125/349, 125/803, 128/751, 128/765, 130/959, 130/1209, 130/1519, 134/229, 134/523, 136/1315, 136/1577).

Furthermore, forty articles provided descriptions of investigations conducted on specific matrices. This included hair in fourteen articles (105/283, 107/269, 108/285, 109/213, 110/55, 110/159, 116/58, 126/451, 128/53, 129/69, 129/259, 132/65, 134/989, 135/1461), bone marrow in three (125/181, 127/915, 136/123), decomposed bodies and bone in four [110/281, 130/371, 134/1339, 135/457], sweat and/or saliva in four (111/82, 112/213, 114/133, 125/675), formalin-fixed organs in two (107/7, 107/165), vitreous humor in two (114/29, 125/4639, and liver homogenate in one article (127/943).

Additionally, forty articles described various analytical methods employed (104/67, 104/263, 105/105, 105/115, 105/265, 106/288, 107/310, 108/191, 108/244, 109/53, 109/80, 109/150, 111/32, 113/150, 113/229, 119/115, 119/355, 121/259, 122/357, 123/451, 124/161, 125/95, 127/413, 129/269, 131/979, 131/989, 131/1001, 131/1271, 131/1543, 133/109, 133/467, 133/1763, 133/1808, 134/205, 134/2105, 135/1437, 135/1471, 135/1813).

Moreover, twelve articles explored the postmortem stability/degradation of drugs (105/87, 111/1, 111/111, 111/165, 120/83, 122/63, 126/259, 127/69, 129/57, 131/369, 131/1283, 135/223), while eleven delved into the postmortem distribution of substances [104/347, 116/207, 116/216, 116/225, 120/226, 121/303, 124/543, 125/831, 130/519, 131/379, 132/1645]. Finally, six articles were related to the field of toxicogenetics (126/315, 127/395, 127/579, 132/1007, 133/353, 134/2095).

## Identification of unknown bodies

The establishment of this group and the attribution of pertinent articles were tricky. Indeed, the recent evolution of identification methods in different subdisciplines such as forensic age estimation, forensic anthropology, and, of course, forensic genetics make it possible to classify the articles in different categories. We decided then to attribute to this group only the articles that were explicitly inherent to the identification procedures of unknown bodies. Fifty-four articles could be included, among which fifteen were dedicated to the identification of victims who died on occasion of mass disasters (104/339, 107/275, 110/47, 113/43, 113/236, 114/19, 114/259, 120/185, 121/517, 125/637, 127/871, 133/277, 134/637, 134/1419, 136/237), and ten discussed scientific approaches or education/training for disaster victim identification (105/83, 107/152, 107/229, 117/204, 132/1545, 134/493, 134/863, 135/375, 135/1983, 136/1801). The remaining articles are related to identification methods specifically tested on human remains.

## Forensic genetics

Forensic genetics stands out as the most prolific category, boasting a total of 1388 articles during this period. Remarkably, six articles from this category rank among the top 10 most cited articles ever published in the International Journal of Legal Medicine (Table 1). While an in-depth analysis of publications in this field is beyond the scope of this article, it may be considered for a separate publication in the future.

The advent of DNA analysis in legal medicine led to the establishment of numerous groups within national and international societies, pioneering this emerging field. Notably, GEDNAP (108/79, 111/97, 116/199, 118/83) and ISFH/ISFG (105/63, 105/361, 107/159, 110/175, 113/193, 114/305, 117/1, 117/5, 120/191, 122/529, 123/227) emerged as the most active of these groups. The pressing need to define common quality standards prompted forensic geneticists to develop proficiency tests (e.g., 110/273, 122/199, 123/227, 133/659, 134/185, 136/397). These tests emphasized the utmost importance of accuracy, objectivity, reproducibility, and logical reasoning, thereby positively influencing all branches of legal medicine.

The articles in this category encompass various aspects of genetic analysis. Specifically, two hundred forty-four articles focused on Autosomal Short Tandem Repeats (STRs), with two hundred nine articles related to Y-chromosome STRs and ninety-seven to X-chromosome STRs. Moreover, ninety-one papers provided information on mitochondrial DNA (mt-DNA). Single Nucleotide Polymorphisms (SNPs) featured in one hundred twenty-six articles, while Insertion-Deletion Polymorphisms (INDEL/DIP) were discussed in thirty-nine articles.

In recent years, microhaplotypes gained attention and were the subject of focus in ten articles (132/703, 133/719, 133/731, 133/983, 135/13, 135/1137, 135/1151, 135/2189, 136/43, 136/1211). The introduction of next-generation sequencing into the forensic context allowed for its application not only in the field of forensic genetics but also in investigating the cause of death (i.e., 129/793, 130/91, 132/1273). This technological advancement has expanded the horizons of forensic genetics (130/905, 131/73, 132/125, 132/1247, 133/219, 133/325, 133/677, 133/1641, 134/1291, 134/2029, 135/1137, 135/1425, 135/1717, 135/2295, 136/447, 136/465, 136/483, 136/671).

## Scientific-technical criminalistics

This category comprises one hundred thirty-two articles. Eleven of them pertain to technical investigations involving sharp forces or instruments (123/129, 125/745, 126/19, 126/43, 131/465, 132/229, 134/1133, 134/1501, 135/2117, 136/329, 136/603). Firearms are the subject of forty-six articles, exploring aspects such as the origin and distribution of backspatters (125/617, 126/391, 129/1027, 133/1839, 135/2061) and conducting tests using simulants (references: 126/607, 131/1043, 132/519, 133/163, 133/1443, 134/309, 135/909). Crime scene investigation takes the center stage in fifteen articles, delving into various aspects of this crucial process (117/170, 124/511, 124/387, 127/723, 127/1093, 130/1379, 131/1119, 132/1067, 133/759, 134/171, 135/1385, 136/729, 136/687, 136/1037, 136/1717). Fourteen articles are dedicated to the analysis of bloodstains (111/17, 126/739, 127/251, 129/139, 130/563, 130/649, 130/731, 131/319, 131/955, 132/875, 132/1625, 133/3, 133/1576, 136/297). The identification and collection of evidence are explored in thirteen papers, discussing essential practices and techniques (118/122, 129/37, 130/599, 131/1413, 132/67, 132/83, 132/683, 132/1025, 133/751, 133/1567, 134/845, 134/1591, 136/1541). Six articles delve into DNA transfer mechanisms, while five focus on bite marks (125/727, 129/709, 131/121, 132/117, 132/373, 132/1035). Additionally, there are two articles on fingerprinting (122/77, 127/85), one on ear prints (119/335), and one on lip prints (127/521).

## Clinical legal medicine

Within this category, we find forty-nine articles. Ten of these articles tackle child abuse from different angles (105/53, 120/73, 124/49, 125/45, 127/627, 129/1091, 133/641, 134/1141, 135/509, 135/1537), with an additional four specifically focusing on abusive head trauma (135/1481, 136/393, 136/591, 136/1009). Sexual abuse is a prominent theme in fourteen papers, encompassing five articles that examine drug-facilitated sexual assaults (120/241, 123/213, 123/155, 126/637, 130/1530). Two studies from Hannover and Turin are included (124/227, 131/1449), alongside five papers addressing sexual abuse in children (112/324, 127/967, 129/153, 131/185, 136/623). Six studies explore violence against migrants and asylum seekers, shedding light on a pressing societal issue (131/1719, 132/1197, 133/669, 134/1495, 135/693, 135/2489). An additional seven papers delve into the subject of torture (117/365, 128/243, 133/297, 135/583, 135/395, 135/2145, 136/391), while elder abuse is discussed in three articles (115/90, 133/317, 135/1515). Two papers provide insights into life-threatening assessments (135/861, 185/871).

## Forensic psychiatry and psychology

Two articles in this category delved into the phenomenon of suicides. More in detail, one publication (107/306) examined the reliability of the Finnish national register of medicolegal autopsies as an epidemiologic tool for suicides. At that time, the autopsy rate for definite suicides was 99% in Finland. Another article explored the epidemiology of suicides in Hungary (108/150). Additionally, there was a report on two cases of transient global amnesia in legal proceedings that discussed its pathogenesis and triggers (129/223).

## Sexual medicine

The author of the single article in this category described a retrospective investigation on 186 expert-appraised pedophile sexual delinquents (111/133).

## Traffic medicine

A total of twenty articles were dedicated to this topic (106/169, 108/265, 122/235, 126/71, 126/357, 128/59, 129/85, 129/471, 129/741, 129/751, 129/997, 129/1011, 130/711, 130/393, 130/405, 130/711, 130/1527, 133/1411, 136/1121, 136/1281). Seven of these articles reported the results of various experiments simulating conditions such as sleep, drugs, and alcohol that may impair an individual's fitness to drive (111/120, 129/471, 129/751, 129/1011, 130/711, 133/1411, 136/1281). Four papers described toxicology methods aimed at assessing fitness to drive (108/265, 122/235, 130/393, 130/1527). Additionally, there were case reports and case series described in six articles (128/59, 129/85, 129/741, 129/997, 130/405, 136/1121). The results of two Swiss surveys on physicians’ knowledge about fitness to drive and medical reporting of unfit drivers were presented in two articles (126/357, 126/71). This category also included a review of the effects of low blood alcohol concentration (BAC) on fitness to drive and international legislation (106/69).

## Social medicine

None of the published papers could be categorized under social medicine.

## Criminology

Six articles were included in this category (131/1055, 132/897, 133/1251, 133/1295, 134/1195, 134/1511). Two articles from a French group reported data on the social and health conditions of arrestees in Paris, with the second article focusing on adolescent arrestees (132/897, 133/1251). Two articles investigated the phenomenon of violence against women in Italy (133/1295) and Montenegro (134/1511). One paper from a German group investigated the relevance of medicolegal reports in criminal investigations in cases of suspected child abuse (131/4). Additionally, a Danish study explored the reliability of police reports concerning health information in cases of medicolegal autopsy (134/3).

## Age estimation

Age estimation is one of the most extensively researched topics in recent years, with a total of two hundres eighty-six articles dedicated to this subject. These articles encompass various aspects of age estimation methods and techniques. Notably, ninety-five of these articles focus on age estimation using dental data, while twenty articles delve into age estimation through the analysis of clavicle bone (118/5, 119/142, 120/15, 121/463, 122/163, 123/241, 128/183, 128/523, 129/187, 129/1259, 130/213, 130/511, 130/1343, 130/1603, 131/217, 132/629, 132/1749, 134/355, 134/753, 136/1017).

Additionally, eighteen articles explore age estimation based on pelvic structures (125/271, 127/473, 127/825, 130/809, 130/1143, 131/501, 132/279, 132/333, 132/609, 132/1447, 133/603, 133/909, 134/2275, 134/1843, 135/929, 135/1923, 136/785, 136/1637), and 7 articles investigate knee-related age estimation (127/839, 129/603, 130/501, 130/1129, 133/205, 133/1191, 135/631). Furthermore, five articles are dedicated to age estimation in the context of child pornography (127/467, 128/649, 129/621, 129/833, 131/1385), while an additional five articles propose the application of artificial intelligence as a potential approach to age estimation (135/649, 135/665, 135/1589, 136/797, 136/821).

Genetic approaches are a prominent theme, with fifteen articles (117/232, 129/237, 132/1, 132/353, 133/1333, 134/451, 134/721, 134/953, 134/2215, 135/167, 135/1225, 135/2163, 135/2209, 136/987, 136/1655) exploring the use of genetic markers in age estimation. Ethical considerations surrounding forensic age estimation are addressed in four articles (123/199, 128/515, 129/1271, 132/815). Additionally, forty-five articles are dedicated to presenting, validating, or comparing various age estimation methods. Among these, significant attention is given to methods proposed by the Schmeling’s group (118/5, 119/142, 122/457, 124/321, 126/923, 127/473, 127/691, 128/183, 129/203, 129/583, 130/1615, 131/585, 132/617), Cameriere (120/49, 125/315, 127/825, 132/1151, 134/783, 136/1685, 137/1117), Gustafson (119/22, 126/615, 131/569, 133/921), and Kvaal (123/123, 126/883, 132/1161, 136/269). Recommendations related to age estimation are presented in two papers (114/83, 122/457).

## Forensic anthropology

The field of forensic anthropology has seen a significant increase in relevance within the medicolegal community over the past three decades. This rise in prominence led to the creation of a dedicated section, the Forensic Anthropology Society of Europe (FASE), within the International Academy of Legal Medicine during the 19th IALM meeting held in Milan in September 2003. The establishment of FASE was driven by Eric Baccino, who announced its creation in the newsletter of the IALM published in this journal (118/N1).

In November 2007, an editorial by Bernd Brinkmann (121/431) introduced Issue 5 of Volume 121, which featured 12 articles related to forensic anthropology. A total of one hundred eighty-nine articles have been attributed to this category, reflecting the growing importance of this discipline. These articles cover various aspects of forensic anthropology, including sex estimation of skeletal remains (sixty-seven articles), age-at-death estimation (thirty-seven articles), and stature estimation (ten articles).

A notable focus in this field is facial and cranial reconstruction for identification purposes, with fourteen articles dedicated to this topic (107/209, 108/194, 121/469, 123/351, 125/301, 127/505, 127/699, 129/227, 129/385, 130/533, 130/863, 132/923, 135/2509, 136/1697). Furthermore, six articles explore toolmarks on bones (132/643, 134/543, 134/613, 135/801, 136/329, 136/343), and five are related to virtual anthropology (130/1315, 131/1155, 133/1903, 135/939, 136/1189).

Forensic anthropology is a field ripe for the application of artificial intelligence. Notable contributions include research by Ortega et al., who investigated machine learning and deep learning methods for sex estimation of infants based on images of the ilium (135/2569), and Toneva et al., who proposed a machine learning approach to estimate sex based on cranial measurements (135/951).

## Forensic imaging

Within the domain of forensic imaging, we have identified a total of one hunfred fifty pertinent papers. These publications explore various investigative techniques, with computed tomography (CT-scan) as the primary focus in ninety-eight articles. Seven of these articles provide a comparison between CT-scan findings and autopsy results (120/124, 128/957, 128/987, 130/191, 130/1081, 130/1089, 134/1457).

Furthermore, thirteen articles delve into the critical role of CT-scan in examining gunshot wounds (122/1, 122/441, 124/613, 125/245, 126/37, 127/419, 130/819, 130/1257, 133/1149, 133/1869, 134/1103, 135/829, 135/1889). Additionally, blunt force injuries are investigated in nine papers (113/33, 127/263, 127/1045, 131/731, 131/489, 134/625, 134/1167, 135/2653, 136/1379), while sharp force injuries are discussed in four articles (136/1417, 128/151, 132/463, 134/691), asphyxia in two (126/641, 134/1441), and drowning in two (129/159, 133/181).

Furthermore, CT-scan is proposed as a valuable tool for identification purposes in twenty articles (120/165, 121/507, 122/301, 122/471, 124/257, 124/259, 127/653, 128/235, 129/877, 130/575, 131/1455, 133/1159, 133/1895, 134/1897, 134/637, 134/1957, 135/1015, 135/1879, 135/1993, 136/1067). Additionally, CT-scan is employed for estimating the age of bone fractures in two articles (127/1139, 135/1913).

Four papers present the results of investigations that utilize both CT-scan and MRI (119/129, 130/1061, 134/669, 134/1817). MRI is the subject of discussion in fourteen articles, with seven articles focusing on its application in the broader context of death investigation (110/1071, 129/1127, 130/1003, 131/1369, 132/541, 134/679). Two articles address MRI in relation to asphyxia (121/115, 135/921), two pertain to gunshot wounds (130/457, 131/1363), two focus on blunt force injuries (123/221, 129/317), and one addresses the estimation of postmortem interval (132/1735).

In angiography, a subject explored in twenty-eight articles, postmortem CT angiography (PMCTA) is the predominant investigative method, featuring in twenty articles. Three of these papers compare the results of PMCTA with those obtained through autopsy (127/639, 127/981, 132/249), while one paper compares PMCTA to histological analysis (127/809). An additional fifteen articles evaluate the performance of PMCTA in detecting vascular lesions (106/55, 112/107, 114/163, 125/609, 127/661, 127/991, 129/1067, 129/1253, 130/441, 130/469, 130/759, 132/589, 135/913, 135/1869, 136/245). MRI angiography is discussed in two papers (131/739, 132/579). Lastly, 3D scanning emerges as investigative method in eight articles, primarily applied for documentation of injuries, identification, or reconstruction (125/785, 126/89, 131/751, 132/551, 132/1241, 133/1167, 136/209, 136/1391).

## Forensic entomology

With the editorial (118/187), Bernd Brinkmann opened issue 118 in which ten paper dealt with forensic entomology, at that time (2004) still an emerging field. The examination of flies (Diptera) is a prominent theme, with 40 papers dedicated to these insects, notably featuring thirty papers focusing on blow flies (Calliphoridae). Coleoptera, another insect group, is described in eleven papers (123/103, 123/285, 128/207, 128/1021, 130/273, 132/887, 132/939, 132/1795, 133/1549, 134/1531, 134/1963).

Guidelines and best practices for forensic entomology were outlined by Amendt et al. (121/90), underlining the need for standardized procedures in this field. Subsequently, Bacqué and Amendt emphasized the importance of validation in court proceedings (127/213).

Entomotoxicology, a specialized branch, is the subject of six papers (114/197, 118/190, 118/194, 118/210, 131/1299, 131/1399). Noteworthy is the retrospective study by Lutz et al. (135/2637), which examined nine hundred forty-nine cases, further underscoring the increasing significance of entomotoxicology in forensic investigations.

## Discussion

Complexity is probably the term that best describes the evolution of medicolegal sciences in the last three decades. It has often been tricky to categorize articles into a specific discipline, as many of them could be attributed to more than one category, depending on the angle of evaluation. For example, imaging techniques (forensic imaging) may be used for detecting vital reactions (general legal medicine) in skeletal remains (forensic anthropology) or to assess the age of a migrant (age estimation). This example can be multiplied by mixing up numerous different investigation strategies and aims. Therefore, the distribution of the articles that we propose is based on the main aspect emerging from each publication; this approach necessarily reflects the authors' opinion.

The first result worth commenting on is the number of publications: four thousand ans four papers were published in IJLM in the considered period, corresponding to 53% of all the articles published in IJLM since its launch. The mean number of articles published in each issue during this period was one hundred twenty-one. In comparison, it was forty-six between 1922 and 1944, thirty-three between 1948 and 1969, and thirty-nine between 1970 and 1990 (volume 103). Landhuis [5] already indicated in 2016 that the number of published scientific papers had climbed by 8–9% per year over the past decades and that about two papers per minute were published in PubMed in the biomedical field alone. Our observations align perfectly with these data.

Table 3 compares the distribution of articles in different categories over four periods. The categories: history, personalia, legal issues, forensic psychiatry, sexual medicine, social medicine, and criminology collectively accounted for less than 1% of publications between 1990 and 2022, whereas articles attributed to the same categories constituted 23.3% during the period 1922–1944. This shift is likely a result of the increased specialization observed in all medical disciplines in recent decades. Articles in fields less closely related to classical legal medicine have progressively migrated towards other more specialized journals. Consequently, IJLM has also evolved into a more specialized publication.

Conversely, we observed a significant increase in papers in the field of forensic genetics (36.9%). Many of these articles presented the frequencies and distribution of various markers in different populations. The publication of this data became necessary due to the growing complexity of caseworks faced by forensic laboratories worldwide. One reason for this complexity is the migration phenomenon that has made Western societies increasingly multiethnic, requiring the identification of individuals or traces in this emerging context. Furthermore, over the last three decades, medicolegal scientific communities in Asia, Central and South America, and Africa have become more active, resulting in the publication of scientific data from these regions in IJLM, significantly broadening the journal's international horizons.

Four new subdisciplines were introduced in this analysis: forensic age estimation (7.6%), forensic anthropology (5.1%), forensic imaging (4%), and forensic entomology (2.4%). The increasing interest of the medicolegal scientific community in these fields is evident. However, the reasons for this growing interest are less clear. A significant portion of the papers in these categories could have been attributed to other subdisciplines already listed in the first three articles of this series. For example, anthropology papers aimed at discriminating sex, estimating age-at-death, or stature could ultimately fall under the category 'Identification of unknown bodies.' Similarly, many papers reporting DNA investigations aimed at identifying human remains could be included in this category. While this would be a technically correct but superficial analysis, it is likely that, initially, new methods were tested to address existing questions. However, over time and with increasing experience, scientists began to explore other innovative applications, creating new avenues of development. A notable example is forensic entomology, whose initial applications were related to estimating the time of colonization of a cadaver, indirectly allowing for the estimation of the time since death. However, quite soon, forensic entomologists began to explore the potential of insects as surrogates for toxicological analyses in cases of advanced putrefaction [6].

Forensic age estimation has become one of the primary topics published in this journal, with articles related to this area ranking among the most cited. Once again, migration and the specific need to identify and protect unaccompanied minor migrants may explain this development. However, it's important to note that this topic is ethically highly sensitive and susceptible to political instrumentalization. In such a context, the rigorous work carried out by specific societies such as AGFAD and FASE has allowed medicolegal experts to gain the specific competencies and expertise required for this task [7, 8].

The introduction of forensic imaging in the medicolegal context sparked intense debates in the early years of this century. Those who were already in the field at the time will recall spirited discussions between those who considered forensic imaging a revolutionary tool capable of replacing the autopsy and those who, more conservative, did not see the need for such additional, expensive, and time-consuming methods in the autopsy room. Twenty years later, we can objectively observe that no revolution has occurred, and forensic pathologists still perform autopsies. However, many institutes of legal medicine have acquired their own CT scanners or have established collaborations with radiologists to conduct postmortem CT scans before a forensic autopsy. The benefits of forensic imaging as a complement to the autopsy are no longer debated, and current research is now exploring the potential of MRI in postmortem investigations [9]. Imaging techniques have become a common tool in legal medicine, extending beyond the specific context of postmortem investigations. Anthropology and age assessment primarily rely on the interpretation of radiological investigations.

The development of the category clinical legal medicine merits comment. With forty-nine articles, it represents only 1.2% of the papers published between 1990 (volume 104) and 2022. However, when compared to previous periods, there is evident progress in clinical examinations for forensic purposes. Brinkmann et al. [10] emphasized as early as 1994 the importance of this activity as a fundamental component of postgraduate education in legal medicine. Over time, the field has evolved towards increased protection of vulnerable individuals, leading forensic physicians to be increasingly involved in investigations on living victims of violence, such as child abuse, sexual abuse, and torture.

In recent years, numerous papers have presented new approaches, exploring the potential of proteomics, metabolomics, neural networks, and deep learning in legal medicine. Testing these newly available methods for age-old but still relevant questions is an intelligent way to strike the necessary balance between relevance and novelty that qualifies a paper for publication. In the coming years, we will see whether these methods become commonplace in medicolegal practice. What is already certain, however, is that classical topics, such as the determination of wound vitality or the estimation of time since death, continue to appear in recent publications.

Classical legal medicine, in contrast, has seen relatively little evolution over the last quarter of a century. While many articles have been published in various fields of classical forensic medicine, these have often been isolated studies, studies with too few cases, or studies on animal models with results difficult to apply routinely. In contrast, forensic toxicologists, geneticists, anthropologists, entomologists, and age estimation experts have worked intensively within their specialist communities to develop a common knowledge base and recommend best practices in their sub-disciplines [e.g., 7, 11–16]. Forensic pathologists have often preferred an individual, patchy approach, often due to specific local opportunities or personal interests. The result is that internationally validated and recognized methods for estimating time since death or determining the age of a skin wound (just to mention two evident examples) simply do not exist. As suggested by Ferrara et al. [17], the solution to this situation is the establishment of multicentric research groups capable of collecting more relevant case numbers and thus publishing more robust results. Meanwhile, national and international medicolegal societies should pool their expertise and resources to produce recommendations in key areas of interest.

The International Journal of Legal Medicine would remain the preferred medium for disseminating these publications.

### Supplementary Information

Below is the link to the electronic supplementary material.

Supplementary file1 (XLS 56 KB)

## Data Availability

Data for this research is easily accessible through the International Journal of Legal Medicine website.
